# FRP-Confined Recycled Coarse Aggregate Concrete: Experimental Investigation and Model Comparison

**DOI:** 10.3390/polym8100375

**Published:** 2016-10-21

**Authors:** Yingwu Zhou, Jingjing Hu, Mali Li, Lili Sui, Feng Xing

**Affiliations:** Guangdong Provincial Key Laboratory of Durability for Marine Civil Engineering, Shenzhen University, Shenzhen 518060, China; ywzhou@szu.edu.cn (Y.Z.); hujingjing@email.szu.edu.cn (J.H.); Maliter@outlook.com (M.L.); suill8969@163.com (L.S.)

**Keywords:** fiber-reinforced polymer (FRP), recycled aggregate concrete (RAC), FRP confined RAC, stress–strain relationship

## Abstract

The in situ application of recycled aggregate concrete (RAC) is of great significance in environmental protection and construction resources sustainability. However, it has been limited to nonstructural purposes due to its poor mechanical performance. External confinement using steel tubes and fiber-reinforced polymer (FRP) can significantly improve the mechanical performance of RAC and thus the first-ever study on the axial compressive behavior of glass FRP (GFRP)-confined RAC was recently reported. To have a full understanding of FRP-confined RAC, this paper has extended the type of FRP and presents a systematic experimental study on the axial compressive performance of carbon FRP (CFRP)-confined RAC. The mechanical properties of CFRP-confined RAC from the perspective of the failure mode, ultimate strength and strain, and stress–strain relationship responses were analyzed. Integrated with existing experimental data of FRP-confined RAC, the paper compiles a database for the mechanical properties of FRP-confined RAC. Based on the database, the effects of FRP type (i.e., GFRP and CFRP) and the replacement ratio of recycled coarse aggregate were investigated. The results indicated that the stress–stain behavior of FRP-confined RAC depended heavily on the unconfined concrete strength and the FRP confining pressure instead of the replacement ratio. Therefore, this study adopted eleven high-performance ultimate strength and strain models developed for FRP-confined normal aggregate concrete (NAC) to predict the mechanical properties of FRP-confined RAC. All the predictions had good agreement with the test results, which further confirmed similar roles played by FRP confinement in improving the mechanical properties of RAC and improving those of NAC. On this basis, this paper finally recommended a stress–strain relationship model for FRP-confined RAC.

## 1. Introduction

The increasing amount of waste concrete has been bringing far-reaching effects on ecological environment deterioration. However, the waste concrete can be processed into recycled coarse aggregate which, serving as a partial or full substitute for natural aggregate, can then be mixed with other ingredients to further produce recycled aggregate concrete (RAC) [1]. The in situ applications of RAC can not only solve the problem of environmental pollution caused by waste concrete, but also relieve the pressure of the scarcity of natural aggregate resources and benefit the sustainable development of the construction industry. However, due to the defects associated with recycled aggregates, such as high porosity, high water absorption rate, and high crushing value, concrete prepared with recycled aggregate has a low strength, and particularly, its durability properties are all inferior to those for natural aggregate concrete (NAC) [1]. The apparent contradictions among mechanical properties, durability, and sustainability have limited the promotion and application of RAC.

Extensive research has shown that using fiber-reinforced polymer (FRP) to effectively confine concrete can significantly improve the mechanical properties of NAC [2,3,4,5]. The effective lateral confinement of FRP places the core concrete in a triaxial compressive stress state, and therefore, can highly increase the ultimate stress of concrete in a confined system, providing an effective approach to overcome the defects of RAC. A large amount of studies on FRP-confined NAC have been conducted during the past two decades. Many studies focused on circular confined sections where concrete is subjected to an in-plane hydrostatic confinement action. Fewer studies have concerned square and rectangular sections, where it is well known that the confinement effect is reduced, since the confinement action is not hydrostatic (in the plane) any more [6,7,8,9,10] and the effect of the corner radius has to be properly considered [4,9,11], together with the cross sectional aspect ratios [12]. In addition, existing studies have also investigated the effects of the following key variables: concrete strength [2,3,9,13,14], fiber type [5,15,16,17,18,19], concrete deterioration [9,20,21], slenderness ratio [22], load eccentricity[23,24,25], size effect [26,27,28], and internal steel reinforcement [9,18,27,28,29]. Further on, it is worth noticing that (1) FRP can be combined with steel profile [30,31]; (2) FRP can be in the form of continuous composite sheets, tapes, or spirals [32,33,34,35]; (3) FRP can be partially or fully wrapped onto the external surface of a column [27,36,37]; and (4) FRP can be considered efficient for existing as well as new constructions, where the concrete columns can be conceived as composite columns combining reinforced concrete core with FRP tube that could be used as formwork. The extensive studies also resulted in a wide range of stress–strain relationships that developed for FRP-confined NAC [5,13,38,39,40,41,42,43,44,45,46,47,48]. The results of these studies have certainly enhanced the understanding of the behaviors of FRP-confined NAC. However, only few researches have focused on studying the FRP-confined RAC, and these studies are mainly limited to glass FRP (GFRP)-confined RAC. For instance, Xiao et al. [49] explored the influence of the replacement ratio of recycled aggregate on the axial and eccentric compressive behaviors of GFRP tube-confined RAC, and the results indicated that with the increase of the replacement ratio of recycled aggregate, the peak strength of GFRP tube-confined RAC declined, accompanied by an increase in the ultimate strain. Xiao et al. [50] compared the differences in the mechanical properties between GFRP tube-confined RAC and steel tube-confined RAC and proposed a stress–strain relationship based on the experimental results and finite element simulations. Zhao et al. [51] conducted the first axial compressive test on GFRP-confined RAC and observed that the replacement ratio of recycled aggregate has limited effect on the compressive behavior of GFRP-confined RAC. They also compared two existing stress–strain models developed for FRP-confined NAC with their test results and concluded that these models could provide a reasonable initial approximation of the stress–strain behavior. Most recently, Chen et al. [52] reported the first ever study on the behavior of carbon FRP (CFRP)-confined RAC, following the same method and procedure as that carried out by Zhao et al. [51] and drawing similar conclusions.

Despite the useful applications that FRP-confined RAC can have, the above discussion indicates that only few studies are present in literature and these studies are mainly limited to glass GFRP-confined RAC, so that, as first step, this paper first presents an experimental study on the axial compressive behaviors of CFRP-confined RAC. Considering the existing data of FRP-confined RAC [51,52], the influences of the replacement ratio of recycled coarse aggregate (i.e., 0%, 20%, 25%, 30%, 50%, 75%, and 100%) and the types of FRP used for the lateral confinement, were analyzed. Eleven widely-recognized strength and strain models developed for FRP-confined NAC were used to predict the experimental results of FRP-confined RAC to clarify possible differences between FRP-confined NAC and RAC, based on which a stress–strain relationship model for FRP-confined RAC was recommended. In view of the social, environmental, and economic significance of RAC, the possibility of using FRP as external tube can be considered particularly appealing if it is combined with an RAC concrete in the framework of so-called “ecofriendly” applications, not excluding the possibility of using recyclable composite tapes and ropes [53] to confine RAC. The achievements of this paper can thus serve as a theoretical basis not only for the application of FRP to strengthen RAC structures but also for the design of RAC composite structures.

## 2. Experimental Program

This paper first performed an axial compressive test of CFRP-confined RAC. The specific experimental methods are described in this section.

### 2.1. Recycled Aggregate (RA)

**Figure 1:**
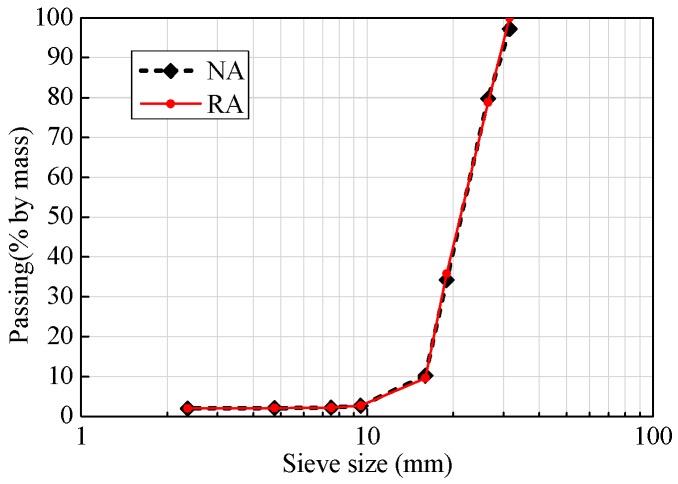
Gradation curve of Recycled Aggregate (RA).

The RA specimens used in the test were prepared using local construction concrete wastes and mixed with specific ratios after crushing, cleaning, and grading to produce RAC. Given that the properties of RA are different from those of natural aggregate (NA), this paper firstly determined and compared the major properties of the two types of aggregates, as shown in Table 1, where the test results of their apparent density, water absorption rate, stacking density, and crushing index were compared. A gradation test was also conducted for the two types of aggregates, and the results are shown in Figure 1.

### 2.2. Design of Test Specimens

Test specimens consisted of forty-eight RAC cylinders. All the specimens had a diameter of 150 mm and a height of 300 mm. The specimens were divided into three series based on the concrete strength, replacement ratio of RA, and number of externally-wrapped CFRP layers, as shown in Table 2. Series C1, C2, and C3 in this table correspond to the three types of RAC with three different mix proportions, as shown in Table 3. It should be noted that the behavior of FRP-confined RAC depends heavily on the unconfined strength of RAC [51,52], as well as the effects of the water absorption that, not being independent, were not considered individually, but had been included in the study of the replacement ratio of RA. Therefore, series C1 was designed to only study the effect of replacement ratio of RA, so the mix proportion was fixed, i.e., the water-to-cement (W/C) ratio, the total dosage of water, sand, and coarse aggregates remained the same, but the composition of the coarse aggregates changed depending on the replacement ratio of RA (i.e., 0%, 30%, 50%, and 100%). Series C2 and C3 were designed to investigate the behaviors of CFRP-confined RAC with different concrete strength but the same replacement ratio of RA (i.e., 100%). Detailed information on these 48 test specimens is summarized in Table 4, where the specimens are named as follows: (1) the letter C with a single-digit number to identify which series the specimens belonged to; (2) the letter R followed by a number to define the replacement ratio of RA; (3) the letter E with a single-digit number to denote the number of CFRP layers; and (4) the letter N followed a number (1–3) to differentiate the three identical specimens (note that three repeated specimens were conducted for each case). For example, C1R50E3N1 is the first specimen of a group that belonged to series C1 and has a replacement ratio of 50% and a three-ply FRP wrap.

### 2.3. Material Properties

The mix proportions and the 28-day cylinder compressive strength of the three series of RAC are summarized in Table 3, respectively. The CFRP adopted in this study had a single-layer fiber thickness of 0.167 mm with the following measured mechanical properties by flat coupon test: ultimate tensile strength of 3931.5 MPa, elastic modulus of 272.7 GPa, and ultimate elongation of 1.6%. The adhesive used in the tests consisted of special bi-component impregnating adhesive, with the following mechanical properties provided by the manufacturer: ultimate tensile strength of 55.5 MPa, compressive strength of 78.4 MPa, flexural strength of 94 MPa, shear strength of 19 MPa, elastic modulus of 3.215 GPa, and ultimate elongation of 2.2%.

### 2.4. Specimen Preparation

The cast specimens were cured in standard condition until the age of 28 days. After curing, to provide a better condition for bonding CFRP, a high-pressure air rifle was used to remove the attached dust and a cement paste was filled into the holes on the surface with a large diameter due to poor condition of concrete pouring; however, resin/primer was used as filler if only small holes existed. The adhesive made by mixing compositions A and B in a weight ratio of A:B = 2:1 was first uniformly applied to the surface of specimens and one or three layers of pre-impregnated CFRP was then attached along the circumferential direction of the specimens with an overlap length of 150 mm. Meanwhile, two layers of CFRP with a width of 50 mm were additionally wrapped at both ends of the specimens to avoid premature rupture of the FRP due to the stress concentration at the ends. The overlap length was 150 mm as well. The ends of specimens were polished to ensure a smooth loading surface before loading.

### 2.5. Test Setup and Instrumentation

**Figure 2:**
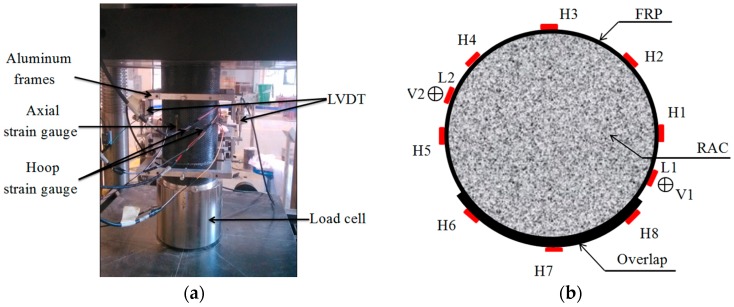
Test setup and instrumentation. (**a**) Test setup; (**b**) Instrumentation.

All the test specimens were loaded by a 3000 kN microcomputer-controlled electro-hydraulic servo pressure machine with displacement control at a loading rate of 0.2 mm/min. The test setup and instrumentation are shown in Figure 2. In Figure 2b, eight strain gauges in the hoop direction, denoted by H1–H8, were installed to obtain the hoop strain distribution of CFRP, and two strain gauges in the axial direction, represented by L1–L2, were installed at the midheight of the specimen to obtain the axial compressive strain. In addition, two linear variable displacement transducers (LVDTs), denoted by V1–V2, were used to measure the axial deformation of the 185-mm midheight region of the specimen; they are used to indirectly obtain the mean axial compressive strain of the specimen.

## 3. Test Results and Discussions

### 3.1. Failure Mode

**Figure 3:**
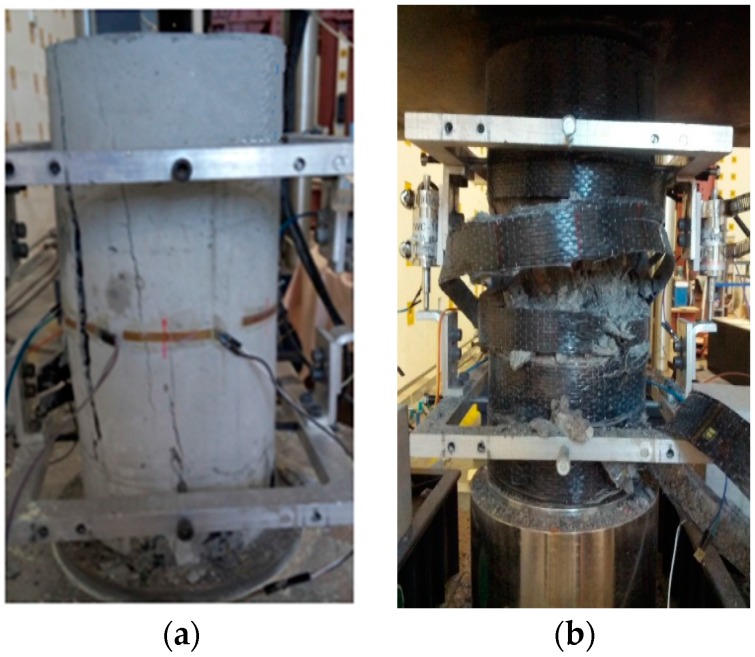
Failure modes. (**a**) Unconfined test specimen; (**b**) Confined test specimen.

A brittle failure was observed in the unconfined RAC test specimens. The test specimen suddenly collapsed when reaching the peak load, the recycled coarse aggregate in the column was cleaved, and multiple longitudinal cracks occurred in the concrete, as shown in Figure 3a. The failure in the CFRP-confined RAC test specimens was caused by the CFRP rupture generally in the middle portion simultaneously accompanied by the collapse of the concrete inside, as shown in Figure 3b, indicating a similar failure mode with the observation by Zhao et al. [51]. Therefore, there is also no noticeable difference in the failure mode between specimens confined with CFRP and GFRP. 

### 3.2. Stress–Strain Behavior

**Figure 4:**
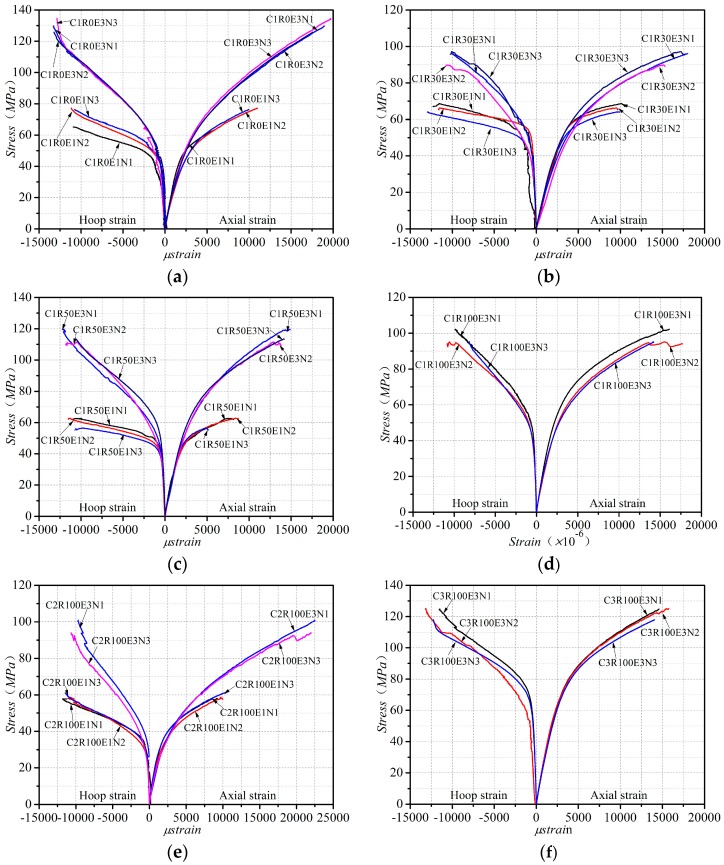
Stress–strain curves of CFRP-confined RAC. (**a**) *R* = 0% in series C1; (**b**) R = 30% in series C1; (**c**) *R* = 50% in series; (**d**) R = 100% in series C1; (**e**) R = 100% in series C1; (**f**) R = 100% in series C3.

**Figure 5:**
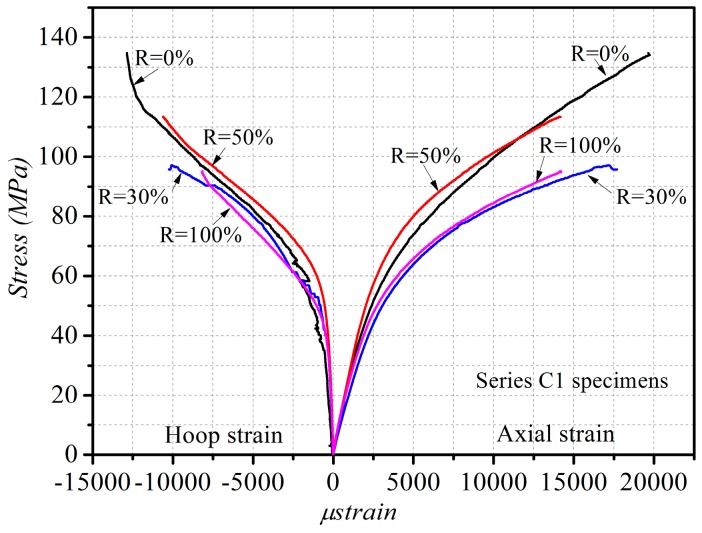
Effect of replacement ratio on stress–strain curve of CFRP-confined RAC (R = 0%, 30%, 50%, 100% in series C1).

The key test results of all the specimens of CFRP-confined RAC are presented in Table 4. Figure 4 shows the axial stress–axial strain curves and axial stress–lateral strain curves of the CFRP-confined RAC, where the axial strain was evaluated by dividing the average values of the two LVDTs by the gauge length of 185 mm, and the hoop strain was averaged from the readings of the five strain gauges outside the overlap area, as shown in Figure 2b. It is observed that during the preliminary stage of loading the CFRP confinement had no significant influence on the stress–strain curves; after the load exceeded the peak stress value of the corresponding unconfined concrete, the CFRP confinement started to constrain the lateral expansion of the core concrete, which resulted in significant improvement of the strength and ductility of RAC. Generally, the axial stress–strain curve of CFRP-confined RAC presented a bilinear characteristic, similar to that of FRP-confined NAC; the ultimate strength and strain of CFRP-confined RAC were enhanced more significantly with the increase of the number of CFRP layers and the unconfined concrete strength. The axial stress–strain curves of CFRP-confined RAC were regrouped in Figure 5, where the specimens in series C1 with different replacement ratio of RA are plotted together and only the stress–strain curve of one of the three identical specimens is provided. It can be seen that, with the increase of the replacement ratio of RA, the transition part between the two portions of the stress–strain curve becomes more circular and the linear portion is getting shorter. In the study of GFRP-confined RAC, Zhao et al. [51] also found a similar effect of the replacement ratio on the curve shape characteristic. 

### 3.3. Hoop Rupture Strain of CFRP

Test results indicate that at the failure of the CFRP-confined RAC, the hoop rupture strain *ε*_h,rup_ of CFRP was significantly less than the CFRP ultimate tensile strain *ε*_frp_ obtained by flat coupon test under uniaxial tensile conditions, as was usually the case for the FRP-confined NAC. The hoop rupture strain *ε*_h,rup_ was determined by averaging the readings from the five hoop strain gauges outside the overlap area, as indicated in Figure 2b. The results are summarized in Table 4. Similar to existing studies on FRP-confined NAC, this paper adopted the ratio *k*_ε_ of the hoop rupture strain *ε*_h,rup_ to the ultimate tensile strain *ε*_frp_ to quantify the efficiency of the CFRP confinement to concrete. The ratio *k*_ε_ is also called the FRP strain efficiency factor. The results are also given in Table 4. From Table 4, it is observed that the CFRP strain efficiency factor *k*_ε_ slightly differs from one specimen to another within the range of 0.596–0.842, with a mean value of 0.709. This value is very close to the mean value of 0.680 for CFRP-confined NAC proposed by Ozbakkaloglu and Lim [48], based on a comprehensive database of FRP-confined NAC. Therefore, it can be concluded that CFRP-confined RAC and NAC have a basically consistent FRP strain efficiency factor, suggesting that the CFRP has a comparable confinement effect on the two types of concrete.

## 4. Influence Factor Analyses

As very limited research has been conducted on the behaviors of FRP-confined RAC, the first ever systematic study on GFRP-confined RAC by Zhao et al. [51] and that on CFRP-confined RAC by Chen et al. [52] were both compared in this section to have a full understanding of the behaviors of FRP-confined RAC. Therefore, the test results of eighteen specimens of GFRP-confined RAC conducted by Zhao et al. [51] and those of forty-seven specimens of CFRP-confined RAC conducted by Chen et al. [52] were all collected and reorganized in Table 5, following the same format as that of Table 4. Considering the present and existing test results as summarized in Table 4 and Table 5, this section will systematically study the influence of the type of FRP and the replacement ratio of RA on the mechanical properties of FRP-confined RAC.

### 4.1. Effects of FRP Type

**Figure 6:**
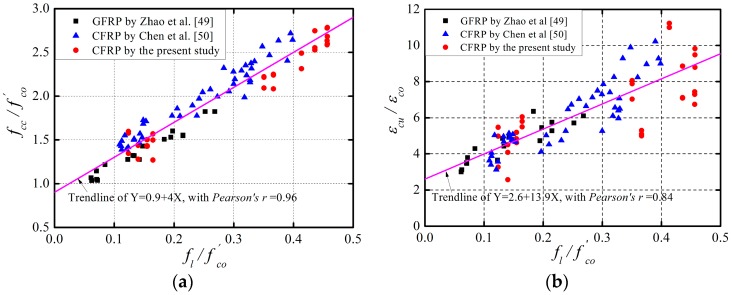
Effects of confinement ratio on FRP-confined RAC. (**a**) Strength gain ratio vs. confinement ratio; (**b**) Strain gain ratio vs. confinement ratio.

As indicated in Figure 4 and Figure 5, the stress–strain behavior of FRP-confined RAC is similar to that of FRP-confined NAC in terms of the curve shape, presenting a noticeable bilinear relationship. CFRP and GFRP impose different confining pressures on concrete and have significantly different effects on the mechanical improvement of the confined concrete [48,54]. Massive studies have adopted the confinement ratio *f_l_*/$f_{co}^{\prime }$ (i.e., the ratio of the hoop confining pressure $f_{l}$ of FRP to the compressive strength $f_{co}^{\prime }$ of unconfined concrete) to quantify the confinement efficiency of the two materials to concrete [2,3,41,42,43,44,45,46,47,48]. Figure 6 presents the relationship between the strength gain ratio $f_{cc}$/$f_{co}^{\prime }$ (i.e., the ratio of the ultimate strength $f_{cc}$ of FRP-confined RAC to the compressive strength $f_{co}^{\prime }$ of unconfined concrete) and the confinement ratio, as well as the relationship between the strain gain ratio *ε_cu_*/*ε_co_* (i.e., the ratio of the ultimate strain *ε_cu_* of FRP-confined RAC to the peak strain *ε_co_* of unconfined concrete) and the confinement ratio. It can be clearly seen that both the strength gain ratio and strain gain ratio exhibit an approximately consistent linear growth trend with the increase of the confinement ratio regardless of the type of FRP, which are further examined by linear regression of the test data. The Pearson correlation coefficients of the fittings were respectively calculated to be 0.96 and 0.84, indicating the high linear relationship. This fully demonstrates that the mechanical properties of FRP-confined RAC significantly depend on the confinement ratio, as is the case for the FRP-confined NAC [2,3,41,42,43,44,45]. It can be concluded that the effect of the FRP type need not to be considered individually as it is fully included in the effect of the confinement ratio.

Table 6 presents the FRP strain efficiency factor of GFRP-confined RAC, with a range of 0.686–0.895 and a mean value of 0.794. This value is also very close to the mean value of 0.793 that is proposed by Ozbakkaloglu and Lim [48] for GFRP-confined NAC based on a comprehensive database, suggesting that GFRP has equivalent confinement efficiencies to RAC and NAC.

### 4.2. Effects of the Replacement Ratio of RA

**Figure 7:**
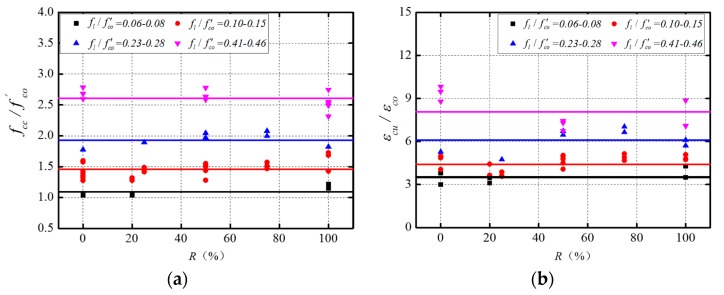
Influence of the replacement ratio of RAs. (**a**) Strength gain ratio vs. replacement ratio; (**b**) strain gain ratio vs. replacement ratio.

As aforementioned, the confinement ratio has covered the possible effect of the FRP type on the mechanical performance of FRP-confined RAC. The effect of the replacement ratio was thus investigated under the same or similar level of the confinement ratio to exclude the potential influence of FRP type. Therefore, the test data summarized in Table 4 and Table 5 were combined to extend the sample space so as to reach more general conclusions. The data with similar confinement ratios but different replacement ratios were reorganized into one group. A total of four groups were thus created, each of which had a very close confinement ratio, as shown in Figure 7, where the influences of the replacement ratio on the ultimate strength and ultimate strain of FRP-confined RAC were respectively examined. As can be observed from Figure 7, for each group of specimens with very close values of confinement ratio, both the strength gain ratio and strain gain ratio were basically the same regardless of the change in the replacement ratio (i.e., 0%, 20%, 25%, 30%, 50%, 75%, and 100%). These values are slightly fluctuating around the mean value line (the horizontal line in Figure 7 represents the mean value of the strength ratio and strain ratio of each group). For each group of data, two statistics, i.e., the mean value and the coefficient of variation (COV), were evaluated to quantitatively assess the variation of the strength gain ratio and strain gain ratio; the results are summarized in Table 6. It is clearly seen that each group of data has significantly small COVs, which indicates that under the same confinement ratio the replacement ratio has limited influence on the mechanical behaviors of FRP-confined RAC, further confirming that the confinement ratio constitutes the primary factor affecting the mechanical behaviors of FRP-confined RAC.

## 5. Comparisons with Existing Ultimate Strength and Strain Models

**Figure 8:**
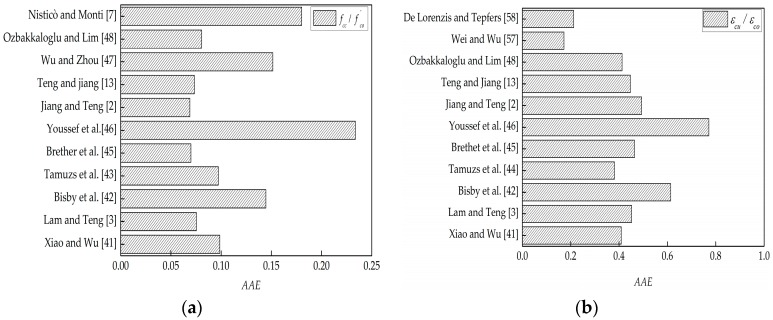
Models performance. (**a**) Ultimate strength models; (**b**) ultimate strain models.

Studies by Zhao et al. [51] and Chen et al. [52] indicated that existing models for FRP-confined NAC could provide a reasonable initial approximation of the stress–strain behavior ofGFRP-confined RAC. Recently, Ozbakkaloglu & Lim [48], Ozbakkaloglu et al. [55], and Nisticò et al. [56] had reviewed and assessed extensive existing FRP-confined NAC models, and to draw a more general conclusion on the difference in behaviors between FRP-confined RAC and FRP-confined NAC, in this section, the eleven best-performing ultimate strength and strain models for FRP-confined NAC recommended by Ozbakkaloglu & Lim [48], Ozbakkaloglu et al. [55], and Nisticò et al. [56] (see Table 7 and Table 8) are selected to predict experimental results from the present and existing studies (see Table 4 and Table 5). Five statistical indexes are respectively adopted to quantitatively assess the possible differences, i.e., the average absolute error (AAE), mean square error (MSE), standard deviation (SD), coefficient of variation (COV), and ratio of prediction to experimental value (RPE), as given by Equations (1)–(5), respectively. Specifically, MSE and AAE were used to assess the overall average deviation of these models while SD and COV were used to reveal the degree of dispersion of the deviations. The evaluations of the five statistical indexes are summarized in Table 9 and Table 10, and Figure 8 shows a comparison on AAE of the eleven models. It should be noted that for the model comparisons, the measured hoop rupture strains were used to predict ultimate strength and strain, if required.

(1) $$AAE=\frac{\sum_{i=1}^{N}\left|\frac{{\mathrm{mod}}_{i}-\exp_{i}}{\exp_{i}}\right|}{N}$$

(2) $$MSE=\frac{\sum_{i=1}^{N}{\left({\mathrm{mod}}_{i}-\exp_{i}\right)}^{2}}{N}$$

(3) $$SD=\sqrt{\frac{\sum_{i=1}^{N}{\left[{\left(\frac{\mathrm{mod}}{\exp}\right)}_{i}-{\left(\frac{\mathrm{mod}}{\exp}\right)}_{avg}\right]}^{2}}{N-1}}$$

(4) $$COV=SD/{\left(\frac{\mathrm{mod}}{\exp}\right)}_{avg}$$

(5) $$RPE=\frac{\mathrm{mod}}{\exp}$$

where “mod” represents the predicted value, “exp” represents the experimental value, *N* represents the total amount of data, and the subscript “avg” represents the mean value.

As indicated in Table 9 and Table 10 and Figure 8, most of the selected models can provide high performance in estimating the ultimate conditions of FRP-confined RAC. The analysis of the AAE, MSE, SD, and COV indexes indicate that the strength models proposed by Teng et al. [13], Brether et al. [45], and Jiang and Teng [2] have the top performance in predicting the ultimate strength, under which AAE, MSE, SD, and COV, respectively, fall within the values of 0.073, 0.030, 0.093, and 0.051, as shown in Figure 8a and Table 8; and the strain models proposed by De Lorenzis et al. [58] and Wei and Wu [57] have the best performance in predicting the ultimate strain, under which AAE, MSE, SD, and COV, respectively, fall below the values of 0.212, 2.624, 0.165, and 0.034, as shown in Figure 8b and Table 10. The results indicate that the replacement ratio of RA does not show a significant effect on the performance of the models in terms of the ultimate strength and strain, and thus existing models developed for FRP-confined NAC can provide an acceptable approximation of the ultimate conditions of FRP-confined RAC, regardless of the type of FRP.

## 6. Comparisons with Existing Stress–Strain Models

### 6.1. Existing Stress–Strain Models and Discussion

**Figure 9:**
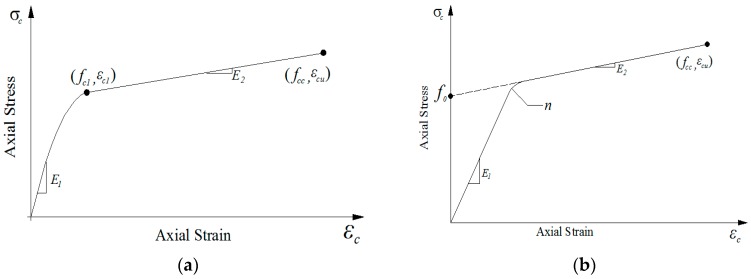
The representative stress–strain models. (**a**) Piecewise function; (**b**) Single function.

The stress–axial strain behavior of FRP-confined RAC is of great importance to developing a good design approach and thus needs to be well understood and properly modeled. As aforementioned, the axial stress–axial strain behavior of FRP-confined RAC is similar to that of FRP-confined NAC, and therefore, existing axial stress–axial strain models developed for FRP-confined NAC are first summarized and then compared with the test results of FRP-confined RAC. In recent years, a series of axial stress–axial strain models related to FRP-confined NAC based on experimental studies and theoretical analyses have been proposed, of which eight representative axial stress–axial strain models are selected for comparison, as shown in Table 11. It can be observed that these models either adopt piecewise continuous functions of parabolas and straight lines [3,13,46,57] (see Figure 9a) or introduce complex single nonlinear functions (see Figure 9b) [59] to describe the axial stress–axial strain curves of FRP-confined NAC. Currently, the most widely used models belonged to the approximately bilinear models adopting the connection of parabolas and straight lines [3,4,5,6,7,8,9,10,11,12,13,57], as shown in Figure 9a. Bilinear stress–strain relationships have a relatively high prediction precision and rationality, and therefore, have contributed to significant development and continuous improvement in this research area. For instance, the bilinear model proposed by Youssef et al. [46] adopts an *n*-order power function to describe the first ascending portion. As for the second type of axial stress–axial strain model, it was first developed by Samaan et al. [59] using a single expression power function with four physical parameters and hereafter referred as a four-parameter model, which is shown in Figure 9b. The model is a single function model, but the complexity of its form has made impossible its functional integration. In the latest study, Zhou and Wu [60] have proposed another four-parameter single function model which, as shown in Table 11, has a curve form consistent with that depicted in Figure 9b. Zhou and Wu’s model [60] has the following advantages when compared with existing models: (1) in comparison to the piecewise function models (e.g., Lam and Teng’s model [3]), this model is expressed by a single continuous function and each of its four parameters has a specific physical meaning; (2) in comparison to the single function models (e.g., Samaan et al.’s model [59]), this model has a simple form, and is integrable and derivable (note that to be integrable is of great importance in solving the sectional internal force of a member with bending effects, e.g., beam and column). As a result, the model proposed by Zhou and Wu [60] has been successfully applied to model the axial stress–axial strain relationship of FRP-confined concrete with initial damage [20,21,61]. Given that, Zhou and Wu’s model [60] is employed herein for the comparison with the experimental axial stress–axial strain behavior of FRP-confined RAC.

It should be noted that in Zhou and Wu’s model [60] (referred to in Table 11 and Figure 9b), *f*_0_ is the stress value at the intersection of the back-extended asymptotical line (see Figure 9b); *E*_1_ is the initial elasticity modulus of FRP-confined concrete and is approximately equal to the elastic modulus of the unconfined concrete *E_c_* [20,21,61], i.e., *E*_1_ = *E_c_*; *E*_2_ is the slope of the asymptotical line of the hardening branch of the axial stress–axial strain curve and can be calculated from the slope of the approximately straight line connected by the two endpoints (0, *f*_0_) and (*ε_cu_*, *f_cc_*) [20,21,61], i.e., *E*_2_ = (*f_cc_* − *f*_0_)/*ε_cu_*; and finally, *ε_n_* = *nε*_0_, where *ε*_0_ = *f*_0_/*E*_1_, and *n* is the shape parameter of the curve that only affects the curvature in the transition zone and is not sensitive to the entire stress–strain curve, and hence for simplicity, an average value of *n* = 1.0 is adopted. 

Based on the discussion in the above section, the best performing models for the ultimate strength and strain respectively proposed by Jiang and Teng [2] and Wei and Wu [57] are herein associated with Zhou and Wu’s model [60] to predict the axial stress–axial strain behaviors and were respectively given by

(6) $$\frac{f_{cc}}{f_{co}^{\prime }}=1+3.5(\frac{f_{l}}{f_{co}^{\prime }})$$

(7) $$\frac{\varepsilon_{cu}}{\varepsilon_{co}}=1.75+12{\left(\frac{f_{l}}{f_{co}}\right)}^{0.75}{\left(\frac{f_{30}}{f_{co}}\right)}^{0.62}$$

Existing models for the parameter *f*_0_ had included the effect of initial damage of concrete [20,21,61], and thus they need to be properly modified to accommodate for NAC or RAC without damage. Based on the work conducted by Samaan et al. [59], the following relationship is found to best fit the results of *f*_0_ as provided in Table 4 and Table 5:

(8) $$f_{0}=0.1+0.94f_{l}+1.02f_{co}^{\prime }$$

**Figure 10:**
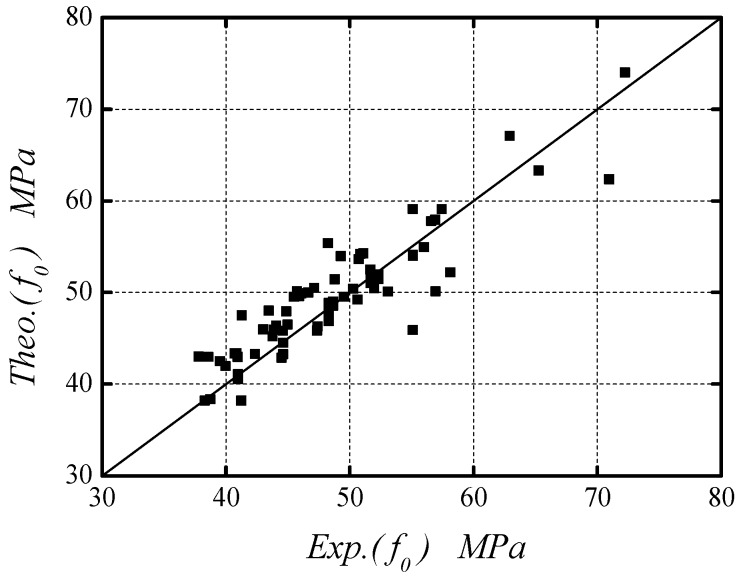
Theo.(*f*_0_) vs. Exp.(*f*_0_).

The performance of Equation (7) is shown in Figure 10, where a mean relative error of 5.31% was evaluated, indicating the reasonable accuracy of the predictions.

The axial stress–lateral stress behavior of FRP-confined RAC is also very crucial to be predicted accurately. Compared with the extensive axial stress–axial strain models, very limited models for the axial stress–lateral strain were available. In this paper, the active-confinement model proposed by Jiang and Teng [2], as summarized in Table 11, is adopted to predict the stress–lateral strain behaviors. It should be noted that Jiang and Teng’s model [2] is also capable of predicting the stress–axialstrain behaviors.

### 6.2. Comparison of Zhou and Wu’s Model and Jiang and Teng’s Model

**Figure 11:**
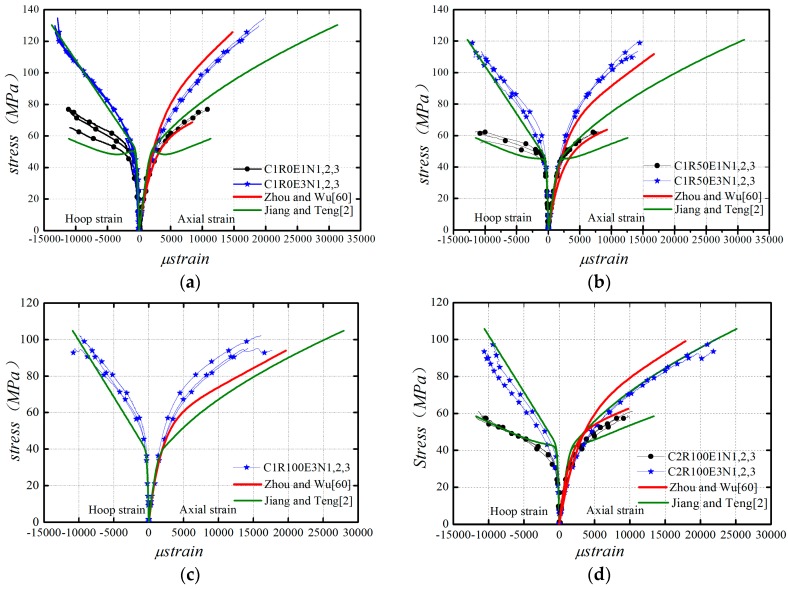

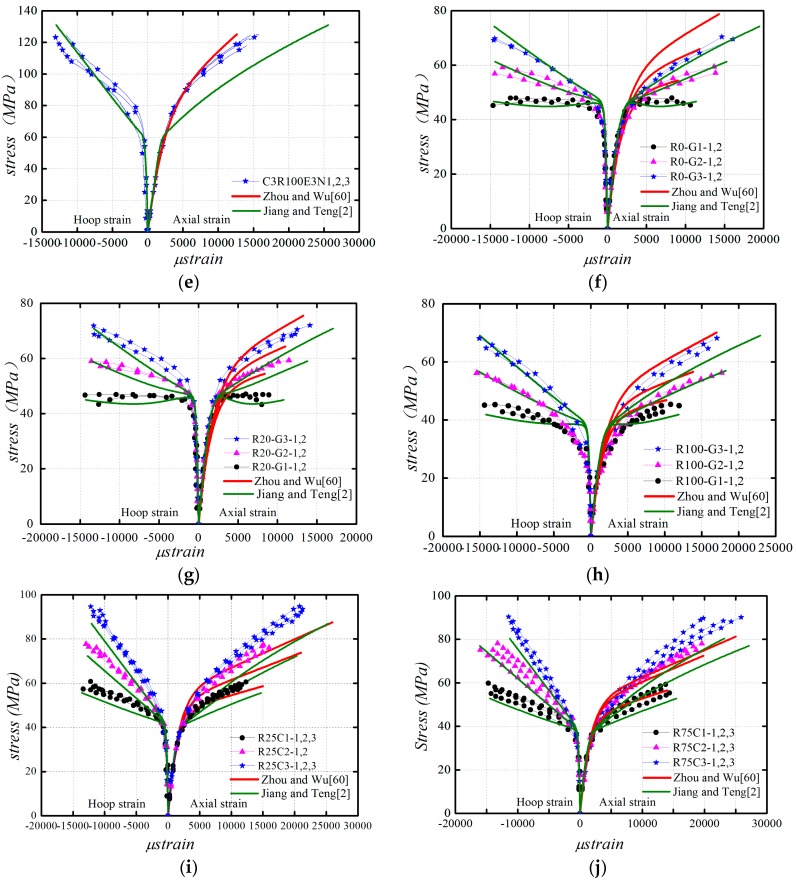
Model evaluations. (**a**) C1R0; (**b**) C2R50; (**c**) C1R100; (**d**) C2R100; (**e**) C3R100; (**f**) R0-G1~3 [51]; (**g**) R20-G1~3 [51]; (**h**) R100-G1~3 [51]; (**i**) R25 [52]; (**j**) R75 [52].

Zhou and Wu’s model [60] combined with the modified model parameters as given by Equations (6)–(8) is used to predict the axial stress–axial strain curves and is compared with the experimental results from the present and existing studies [51,52], as shown in Figure 11. Figure 11 also shows the comparison between the experimental axial stress–axial strain and axial stress–lateral strain curves and the corresponding predictions by Jiang and Teng’s model [2]. The comparisons indicate that Zhou and Wu’s model [60] has a satisfactory agreement with the experimental axial stress–axial strain behavior and Jiang and Teng’s model [2] has a good performance in predicting the axial stress–lateral strain behavior.

## 7. Conclusions

This paper presents a systematic experimental study on the behaviors of CFRP-confined RAC, and combined with the first ever study on the GFRP-confined RAC by Zhao et al. [51] and that on the CFRP-confined RAC by Chen et al. [52], the possible differences between FRP-confined RAC and FRP-confined NAC in terms of ultimate strength and strain, and stress–strain curve, were investigated. The main conclusions obtained from this study are the following:

(1) The compressive behaviors of FRP-confined RAC are similar to those of FRP-confined NAC. Effective FRP confinement can significantly improve the ultimate strength and ultimate strain of RAC.
(2) The experimental results indicate that a different replacement ratio of recycled aggregate between specimens with similar concrete strength (i.e., C2-100% and C1-30%) provide higher ultimate axial strain for higher confinement (see C2R100E3 versus C1R30E3, Figure 4b,e). The analysis of the compiled database for FRP-confined RAC indicates that the mechanical properties of FRP-confined RAC significantly depend on the confinement ratio instead of FRP type and the replacement ratio of RA has a very slight influence on the behavior of FRP-confined RAC.
(3) Through the quantitative assessment of eleven well-recognized strength and strain models of FRP-confined NAC, it can be concluded that the models proposed by Jiang and Teng [2] and Wei and Wu [57] have respectively the top performance in predicting the ultimate strength and ultimate strain of FRP-confined RAC. On this basis, this paper finally recommends the use of Zhou and Wu’s model [60] and Jiang and Teng’s model [2] to respectively simulate the axial stress–axial strain and axial stress–lateral strain behaviors of FRP-confined RAC, which have provided a reasonable accuracy in prediction.

## Figures and Tables

**Table 1:** Properties of coarse aggregate.

Aggregate type	Size range (mm)	Apparent density (kg/m^3^)	Water absorption rate (%)	Stacking density (kg/m^3^)	Crushing index (%)
RA	5–31.5	2476.9	6.31	1,158.2	18.6
NA	5–31.5	2650.0	1.35	1,515.3	11.2

**Table 2:** Specimen details.

Series name	Replacement ratio of RA, *R* (%)	Number of CFRP layers	Number of identical specimens	Subtotal of test specimens
C1	0, 30, 50, 100 ^a^	0, 1, 3	3	33
C2	100	0, 1, 3	3	9
C3	100	0, 3	3	6

^a^ Note that in series C1, the specimen with the replacement ratio of 100% was only wrapped with three layers of CFRP (carbon fiber-reinforced polymer).

**Table 3:** Mix proportions of RAC (recycled aggregate concrete) (kg/m^3^).

Series name	Replacement ratio, *R*	W/C	Cement (kg/m^3^)	Water (kg/m^3^)	RA (kg/m^3^)	NA (kg/m^3^)	Sand (kg/m^3^)	Water reducing agent	Concrete strength (MPa)
C1	0%	0.35	563	190	0	838	1,275	1.80%	48.41
C1	30%	0.35	563	190	251	587	1,275	1.80%	42.74
C1	50%	0.35	563	190	419	419	1,275	1.80%	43.06
C1	100%	0.35	563	190	838	0	1,275	3.50%	37.21
C2	100%	0.45	500	219	880	0	1,259	2.50%	40.54
C3	100%	0.30	625	179	838	0	1,253	3.00%	56.34

**Table 4:** The key experimental results of CFRP (carbon fiber-reinforced polymer)-confined RAC.

Specimen name	*R*	FRP type	*E*_frp_	*f*_frp_	*t*_frp_	FRP layers	$\varepsilon_{co}$	$\varepsilon_{hm,rup}$	$\varepsilon_{h,rup}$ *	$\kappa_{\varepsilon }$	$\varepsilon_{cu}$	*f*_cc_	*E*_c_ *	*f*_o_ *
	(%)		(GPa)	(GPa)	(mm)		(%)	(%)	(%)		(%)	(MPa)	(GPa)	(MPa)
C1R0E1N1	0	CFRP	272.73	3.93	0.167	1	0.2	1.216	1.096	0.685	0.65 ^a^	65.14	30.3	55.08
C1R0E1N2	0	CFRP	272.73	3.93	0.167	1	0.2				1.09	77.28		
C1R0E1N3	0	CFRP	272.73	3.93	0.167	1	0.2				1.00	76.32		
C1R0E3N1	0	CFRP	272.73	3.93	0.167	3	0.2	1.485	1.347	0.842	1.90	129.80	30.3	75.39
C1R0E3N2	0	CFRP	272.73	3.93	0.167	3	0.2				1.96	134.76		
C1R0E3N3	0	CFRP	272.73	3.93	0.167	3	0.2				1.76	125.76		
C1R30E1N1	30	CFRP	272.73	3.93	0.167	1	0.2	1.394	1.285	0.803	1.05	67.04	31.8	42.63
C1R30E1N2	30	CFRP	272.73	3.93	0.167	1	0.2				0.96	65.22		
C1R30E1N3	30	CFRP	272.73	3.93	0.167	1	0.2				0.99	64.04		
C1R30E3N1	30	CFRP	272.73	3.93	0.167	3	0.2	1.101	0.954	0.596	1.80	96.12	31.8	62.93
C1R30E3N2	30	CFRP	272.73	3.93	0.167	3	0.2				1.53	89.03		
C1R30E3N3	30	CFRP	272.73	3.93	0.167	3	0.2				1.77	95.78		
C1R50E1N1	50	CFRP	272.73	3.93	0.167	1	0.2	1.258	1.103	0.689	0.82	61.87	30.8	50.65
C1R50E1N2	50	CFRP	272.73	3.93	0.167	1	0.2				0.90	62.23		
C1R50E1N3	50	CFRP	272.73	3.93	0.167	1	0.2				0.51 ^a^	55.09		
C1R50E3N1	50	CFRP	272.73	3.93	0.167	3	0.2	1.388	1.197	0.748	1.49	119.59	30.8	70.95
C1R50E3N2	50	CFRP	272.73	3.93	0.167	3	0.2				1.35	111.36		
C1R50E3N3	50	CFRP	272.73	3.93	0.167	3	0.2				1.46	113.40		
C1R100E3N1	100	CFRP	272.73	3.93	0.167	3	0.2	1.184	0.988	0.618	1.42	102.19	31.3	65.26
C1R100E3N2	100	CFRP	272.73	3.93	0.167	3	0.2				1.77	94.04		
C1R100E3N3	100	CFRP	272.73	3.93	0.167	3	0.2				1.42	95.01		
C2R100E1N1	100	CFRP	272.73	3.93	0.167	1	0.2	1.283	1.148	0.718	0.92	58.10	31.3	37.82
C2R100E1N2	100	CFRP	272.73	3.93	0.167	1	0.2				0.97	57.80		
C2R100E1N3	100	CFRP	272.73	3.93	0.167	1	0.2				1.04	60.92		
C2R100E3N1	100	CFRP	272.73	3.93	0.167	3	0.2	1.169	1.021	0.638	2.25	101.00	31.3	58.12
C2R100E3N2	100	CFRP	272.73	3.93	0.167	3	0.2				2.20	93.78		
C2R100E3N3	100	CFRP	272.73	3.93	0.167	3	0.2				-	-		
C3R100E3N1	100	CFRP	272.73	3.93	0.167	3	0.2	1.403	1.203	0.752	1.61	124.77	33.0	72.23
C3R100E3N2	100	CFRP	272.73	3.93	0.167	3	0.2				1.58	125.15		
C3R100E3N3	100	CFRP	272.73	3.93	0.167	3	0.2				1.41	117.89		

Note that the superscript lowercase letter “^a^” denotes that the value is excluded due to the large deviation of above 15% from the group average value; “-“ denotes the data is unavailable; and “*” indicates the group average value is summarized.

**Table 5:** Data of FRP (fiber-reinforced polymer)-confined RAC [51,52].

Specimen name	*R*	FRP type	*E_frp_*	*f_frp_*	*t_frp_*	FRP layers	$f_{co}^{\prime }$ *	*ε_co_* *	*ε_h,rup_*	*k_ε_*	*ε_cu_*	$f_{cc}$	*E_c_* *	$f_{o}$ *
	(%)		(GPa)	(GPa)	(mm)		(MPa)	(%)	(%)		(%)	(MPa)	(GPa)	(MPa)
R0-G1-1	0	GFRP	98.7	1.72	0.170	1	45.00	0.280	1.46	0.849	1.06	46.70	30.7	48.67
R0-G1-2	0	GFRP	98.7	1.72	0.170	1			1.24	0.721	0.84	48.00		
R0-G2-1	0	GFRP	98.7	1.72	0.170	2	45.00	0.280	1.33	0.773	1.36	59.50	30.7	52.33
R0-G2-2	0	GFRP	98.7	1.72	0.170	2			1.44	0.837	1.38	57.40		
R0-G3-1	0	GFRP	98.7	1.72	0.170	3	45.00	0.280	1.45	0.843	1.61	69.60	30.7	56.00
R0-G3-2	0	GFRP	98.7	1.72	0.170	3			1.45	0.843	1.48	70.00		
R20-G1-1	20	GFRP	98.7	1.72	0.170	1	44.90	0.260	1.26	0.733	0.81	46.50	31.6	48.30
R20-G1-2	20	GFRP	98.7	1.72	0.170	1			1.43	0.831	0.90	47.10		
R20-G2-1	20	GFRP	98.7	1.72	0.170	2	44.90	0.260	1.24	0.721	0.95	57.30	31.6	51.71
R20-G2-2	20	GFRP	98.7	1.72	0.170	2			1.35	0.785	1.15	59.20		
R20-G3-1	20	GFRP	98.7	1.72	0.170	3	44.90	0.260	1.33	0.773	1.42	71.90	31.6	55.11
R20-G3-2	20	GFRP	98.7	1.72	0.170	3			1.31	0.762	1.23	68.70		
R100-G1-1	100	GFRP	98.7	1.72	0.170	1	37.30	0.280	1.18	0.686	0.98	42.70	27.0	40.97
R100-G1-2	100	GFRP	98.7	1.72	0.170	1			1.42	0.826	1.20	45.40		
R100-G2-1	100	GFRP	98.7	1.72	0.170	2	37.30	0.280	1.24	0.721	1.34	53.30	27.0	44.63
R100-G2-2	100	GFRP	98.7	1.72	0.170	2			1.54	0.895	1.78	56.20		
R100-G3-1	100	GFRP	98.7	1.72	0.170	3	37.30	0.280	1.50	0.872	1.71	68.00	27.0	48.30
R100-G3-2	100	GFRP	98.7	1.72	0.170	3			1.41	0.820	1.60	68.00		
R0C1-1	0	CFRP	250.0	4.517	0.111	1	44.44	0.345	1.34	0.742	1.17	63.56	37.1	54.00
R0C1-2	0	CFRP	250.0	4.517	0.111	1			1.35	0.747	1.40	61.71		
R0C1-3	0	CFRP	250.0	4.517	0.111	1			1.45	0.803	1.08	61.9		
R0C2-1	0	CFRP	250.0	4.517	0.111	2	44.44	0.345	1.18	0.653	1.42	78.94	37.1	49.94
R0C2-2	0	CFRP	250.0	4.517	0.111	2			1.43	0.791	1.82	78.94		
R0C2-3	0	CFRP	250.0	4.517	0.111	2			1.26	0.697	1.56	78.74		
R0C3-1	0	CFRP	250.0	4.517	0.111	3	44.44	0.345	1.31	0.725	2.06	97.04	37.1	56.48
R0C3-2	0	CFRP	250.0	4.517	0.111	3			1.20	0.664	1.82	95.09		
R0C3-3	0	CFRP	250.0	4.517	0.111	3			1.31	0.725	2.21	95.87		
R25C1-1	25	CFRP	250.0	4.517	0.111	1	40.75	0.320	1.24	0.686	1.24	60.64	35.1	44.81
R25C1-2	25	CFRP	250.0	4.517	0.111	1			1.36	0.753	1.14	57.72		
R25C1-3	25	CFRP	250.0	4.517	0.111	1			1.20	0.664	1.16	58.98		
R25C2-1	25	CFRP	250.0	4.517	0.111	2	40.75	0.320	1.27	0.703	1.52	77.18	35.1	48.34
R25C2-3	25	CFRP	250.0	4.517	0.111	2			1.13	0.625	1.61	75.72		
R25C3-1	25	CFRP	250.0	4.517	0.111	3	40.75	0.320	1.18	0.653	2.10	91.2	35.1	50.69
R25C3-2	25	CFRP	250.0	4.517	0.111	3			1.04	0.576	2.28	94.61		
R25C3-3	25	CFRP	250.0	4.517	0.111	3			1.21	0.670	2.08	94.41		
R50C1-1	50	CFRP	250.0	4.517	0.111	1	37.03	0.272	1.18	0.653	1.37	57.43	32.1	39.36
R50C1-2	50	CFRP	250.0	4.517	0.111	1			1.47	0.814	1.30	56.55		
R50C1-3	50	CFRP	250.0	4.517	0.111	1			1.33	0.736	1.35	55.58		
R50C2-1	50	CFRP	250.0	4.517	0.111	2	37.03	0.272	1.46	0.808	2.04	76.11	32.1	45.28
R50C2-2	50	CFRP	250.0	4.517	0.111	2			1.24	0.686	1.83	75.72		
R50C2-3	50	CFRP	250.0	4.517	0.111	2			1.21	0.670	1.76	73.00		
R50C3-1	50	CFRP	250.0	4.517	0.111	3	37.03	0.272	1.16	0.642	2.69	95.09	32.1	48.58
R50C3-2	50	CFRP	250.0	4.517	0.111	3			1.30	0.720	2.78	89.06		
R50C3-3	50	CFRP	250.0	4.517	0.111	3			1.20	0.664	2.24	91.39		
R75C1-1	75	CFRP	250.0	4.517	0.111	1	37.48	0.280	1.35	0.747	1.31	56.65	32.4	39.79
R75C1-2	75	CFRP	250.0	4.517	0.111	1			1.44	0.797	1.44	54.99		
R75C1-3	75	CFRP	250.0	4.517	0.111	1			1.45	0.803	1.37	58.89		
R75C2-1	75	CFRP	250.0	4.517	0.111	2	37.48	0.280	1.38	0.764	1.86	74.75	32.4	43.89
R75C2-2	75	CFRP	250.0	4.517	0.111	2			1.32	0.731	1.97	77.87		
R75C2-3	75	CFRP	250.0	4.517	0.111	2			1.61	0.891	1.71	74.46		
R75C3-1	75	CFRP	250.0	4.517	0.111	3	37.48	0.280	1.13	0.625	2.60	89.84	32.4	45.91
R75C3-2	75	CFRP	250.0	4.517	0.111	3			1.08	0.598	2.31	88.18		
R75C3-3	75	CFRP	250.0	4.517	0.111	3			1.11	0.614	1.98	89.06		
R100C1-1	100	CFRP	250.0	4.517	0.111	1	32.87	0.289	1.32	0.731	1.37	56.74	30.8	39.43
R100C1-2	100	CFRP	250.0	4.517	0.111	1			1.36	0.753	1.35	56.36		
R100C1-3	100	CFRP	250.0	4.517	0.111	1			1.32	0.731	1.47	55.38		
R100C2-1	100	CFRP	250.0	4.517	0.111	2	32.87	0.289	1.34	0.742	2.27	72.12	30.8	42.60
R100C2-2	100	CFRP	250.0	4.517	0.111	2			1.39	0.769	2.14	75.34		
R100C2-3	100	CFRP	250.0	4.517	0.111	2			1.33	0.736	2.11	74.95		
R100C3-1	100	CFRP	250.0	4.517	0.111	3	32.87	0.289	1.11	0.614	2.59	86.63	30.8	44.08
R100C3-2	100	CFRP	250.0	4.517	0.111	3			1.18	0.653	2.60	86.92		
R100C3-3	100	CFRP	250.0	4.517	0.111	3			1.17	0.648	2.68	89.35		
R100C2-3	100	CFRP	250.0	4.517	0.111	2	32.87	0.289	1.33	0.736	2.11	74.95	30.8	42.60
R100C3-1	100	CFRP	250.0	4.517	0.111	3	32.87	0.289	1.11	0.614	2.59	86.63	30.8	44.08
R100C3-2	100	CFRP	250.0	4.517	0.111	3			1.18	0.653	2.60	86.92		

Note that the first 18 rows of data were collected from the work by Zhao et al. [51] while the others were collected from the work by Chen et al. [52]; ”-“ denotes the data is unavailable; and “*” denotes that the group average value is provided.

**Table 6:** Statistical data to quantify the variation of $f_{cc}$***/***$f_{co}^{\prime }$ and *ε_cu_/ε_co_* under different replacement ratios.

H	Group 1			Group 2			Group 3			Group 4		
	$f_{l}/f_{co}^{\prime }$ = 0.06–0.08			$f_{l}/f_{co}^{\prime }$ = 0.10–0.15			$f_{l}/f_{co}^{\prime }$ = 0.23–0.28			$f_{l}/f_{co}^{\prime }$ = 0.41–0.46		
	μ	σ	COV	μ	σ	COV	μ	σ	COV	μ	σ	COV
*f_cc_*/*f ^’^_co_*	1.092	0.074	0.067	1.456	0.121	0.083	1.926	0.112	0.058	2.608	0.140	0.054
*ε_cu_*/*ε_co_*	3.525	0.468	0.133	4.478	0.573	0.128	6.282	0.619	0.099	8.068	1.163	0.144

**Table 7:** Eleven representative ultimate strength models.

No.	Reference	Strength model
1	Xiao and Wu [41]	$\frac{f_{cc}}{f_{co}^{‘}}=1+k_{1}(\frac{f_{lu}}{f_{co}^{’}})$
2	Lam and Teng [3]	$\frac{f_{cc}}{f_{co}^{\prime }}=1+3.3(\frac{f_{l}}{f_{co}^{\prime }})$
3	Bisby et al. [42]	$\frac{f_{cc}}{f_{co}^{\prime }}=1+2.425\frac{f_{l}}{f_{co}^{\prime }}$
4	Tamuzs et al. [43]	$\frac{f_{cc}}{f_{co}^{\prime }}=1+4.2(\frac{f_{l}}{f_{co}^{\prime }})$
5	Brether et al. [45]	$f_{cc}=f_{co}^{\prime }+k_{1}f_{l}$
6	Youssef et al. [46]	$\frac{f_{cc}}{f_{co}^{\prime }}=1+2.25{\left(\frac{f_{l}}{f_{co}^{\prime }}\right)}^{1.25}$
7	Jiang and Teng [2]	$\frac{f_{cc}}{f_{co}^{\prime }}=1+3.5(\frac{f_{l}}{f_{co}^{\prime }})$
8	Teng et al. [13]	$\frac{f_{cc}}{f_{co}^{\prime }}=1+3.5\left(\rho_{k}-0.01\right)\rho_{\varepsilon }$
9	Wu and Zhou [47]	$\frac{f_{cc}}{f_{co}^{\prime }}=\frac{f_{lu}}{f_{co}^{\prime }}+\sqrt{\left(\frac{16.7}{f_{co}^{\prime }}-\frac{f_{co}^{\prime }}{16.7}\right)\frac{f_{lu}}{f_{co}^{\prime }}+1}$
10	Ozbakkaloglu and Lim [48]	$f_{cc}=c_{1}f_{co}^{\prime }+3.26\left(f_{l}-f_{lo}\right)$
11	Nistico and Monti [7]	$\frac{f_{cc}}{f_{co}}=1+2.09\frac{f_{l}}{f_{co}}$

Note that *f_lu_* is defined as the confining pressure and *f_lu_* = 2*E_frp_·t_frp_·ε_frp_*/D, while *f_l_* is called the effective confining pressure and *f_l_* = 2 *κ_ε_·E_frp_·t_frp_·ε_frp_*/D.

**Table 8:** Eleven representative ultimate strain models.

No.	Reference	Strain model
1	Xiao and Wu [41]	$\varepsilon_{cu}=\frac{\varepsilon_{h,rup}+\varepsilon_{0}}{\mu_{tu}}$
2	Lam and Teng [3]	$\frac{\varepsilon_{cu}}{\varepsilon_{co}}=1.75+12\left(\frac{f_{l}}{f_{co}^{\prime }}\right){\left(\frac{\varepsilon_{h,rup}}{\varepsilon_{co}}\right)}^{0.45}$
3	Bisby et al. [42]	$\varepsilon_{cu}=\varepsilon_{co}+k_{2}\frac{f_{l}}{f_{co}^{\prime }}$
4	Tamuzs et al. [44]	$\varepsilon_{cu}=\varepsilon_{co}+\frac{\varepsilon_{h,rup}-\nu_{c}\varepsilon_{co}}{\mu_{tu}}$
5	Brether et al. [45]	$\varepsilon_{cu}=\varepsilon_{co}+\frac{\varepsilon_{h,rup}-\nu_{c}\varepsilon_{co}}{\mu_{tu}}$
6	Youssef et al. [46]	$\varepsilon_{cu}=0.003368+0.2590\left(\frac{f_{l}}{f_{co}^{\prime }}\right){\left(\frac{f_{frp}}{E_{frp}}\right)}^{0.5}$
7	Jiang and Teng [2]	$\frac{\varepsilon_{cu}}{\varepsilon_{co}}=1+17.5{\left(\frac{f_{l}}{f_{co}^{\prime }}\right)}^{1.2}$
8	Teng et al. [13]	$\frac{\varepsilon_{cu}}{\varepsilon_{co}}=1.75+6.5{\rho_{k}}^{0.8}{\rho_{\varepsilon }}^{1.45}$
9	Ozbakkaloglu and Lim [48]	$\varepsilon_{cu}=c_{2}\varepsilon_{co}+0.266{\left(\frac{K_{1}}{f_{co}^{\prime }}\right)}^{0.9}\varepsilon_{h,rup}^{1.35}$
10	Wei and Wu [57]	$\frac{\varepsilon_{cu}}{\varepsilon_{co}}=1.75+12{\left(\frac{f_{l}}{f_{co}}\right)}^{0.75}{\left(\frac{f_{30}}{f_{co}}\right)}^{0.62}$
11	De Lorenzis et al. [58]	$\frac{\varepsilon_{cc}}{\varepsilon_{co}}=1+26.2{\left(\frac{p_{u}}{f\prime_{co}}\right)}^{0.80}{E_{l}}^{-0.148}$

Note that *f_lu_* is defined as the confining pressure and *f_lu_* = 2*E_frp_·t_frp_·ε_frp_*/D, while *f_l_* is called the effective confining pressure and *f_l_* = 2*·κ_ε_·E_frp_·t_frp_·ε_frp_*/D.

**Table 9:** Statistical assessment of the ultimate strength models.

NO.	AAE			MSE			SD			COV			RPE		
	GFRP	CFRP	Both ^a^	GFRP	CFRP	Both ^a^	GFRP	CFRP	Both ^a^	GFRP	CFRP	Both ^a^	GFRP	CFRP	Both ^a^
1	0.083	0.102	0.099	0.012	0.046	0.040	0.038	0.080	0.073	0.030	0.044	0.043	0.917	0.912	0.913
2	0.093	0.071	0.075	0.016	0.033	0.030	0.054	0.070	0.095	0.036	0.037	0.053	1.093	0.949	0.978
3	0.056	0.166	0.145	0.009	0.164	0.133	0.073	0.076	0.109	0.054	0.046	0.069	1.004	0.838	0.871
4	0.184	0.076	0.097	0.065	0.035	0.041	0.044	0.075	0.089	0.027	0.035	0.044	1.184	1.064	1.088
5	0.108	0.061	0.070	0.022	0.024	0.024	0.051	0.070	0.093	0.034	0.036	0.051	1.108	0.968	0.996
6	0.112	0.264	0.234	0.042	0.364	0.301	0.086	0.072	0.109	0.071	0.050	0.078	0.900	0.736	0.769
7	0.113	0.058	0.069	0.024	0.022	0.022	0.050	0.070	0.093	0.033	0.036	0.050	1.113	0.975	1.002
8	0.029	0.084	0.073	0.002	0.037	0.030	0.036	0.080	0.076	0.027	0.042	0.043	0.988	0.943	0.952
9	0.057	0.175	0.152	0.010	0.186	0.151	0.075	0.081	0.116	0.055	0.050	0.074	1.004	0.829	0.864
10	0.096	0.077	0.081	0.018	0.039	0.035	0.055	0.077	0.101	0.037	0.041	0.056	1.096	0.948	0.977
11	0.072	0.207	0.180	0.017	0.248	0.202	0.081	0.080	0.116	0.062	0.051	0.077	0.970	0.795	0.829

^a^ Both = GFRP + CFRP. AAE (the average absolute error), MSE (mean square error), SD (standard deviation), COV (coefficient of variation), and RPE (ratio of prediction to experimental value).

**Table 10:** Statistical assessment of the ultimate strain models.

NO.	AAE			MSE			SD			COV			RPE		
	GFRP	CFRP	Both ^a^	GFRP	CFRP	Both ^a^	GFRP	CFRP	Both ^a^	GFRP	CFRP	Both ^a^	GFRP	CFRP	Both ^a^
1	0.726	0.331	0.409	11.933	9.838	10.252	0.029	0.351	0.371	0.023	0.078	0.096	0.274	0.725	0.636
2	0.673	0.398	0.452	10.539	10.994	10.904	0.039	0.458	0.467	0.026	0.088	0.104	0.327	0.856	0.751
3	0.699	0.593	0.614	11.439	17.524	16.320	0.042	0.119	0.116	0.031	0.048	0.051	0.301	0.407	0.386
4	0.646	0.316	0.381	9.653	9.135	9.238	0.037	0.296	0.302	0.023	0.070	0.081	0.354	0.703	0.634
5	0.668	0.413	0.464	10.388	11.391	11.193	0.038	0.226	0.228	0.025	0.063	0.072	0.332	0.587	0.536
6	0.730	0.782	0.771	12.474	28.714	25.502	0.043	0.064	0.067	0.036	0.050	0.053	0.270	0.218	0.229
7	0.667	0.451	0.494	10.338	11.908	11.597	0.038	0.207	0.207	0.025	0.061	0.069	0.333	0.549	0.506
8	0.704	0.383	0.447	11.318	10.162	10.390	0.030	0.441	0.456	0.022	0.087	0.105	0.296	0.841	0.733
9	0.672	0.348	0.412	10.514	10.365	10.395	0.039	0.291	0.294	0.026	0.073	0.084	0.328	0.661	0.595
10	0.090	0.193	0.172	0.279	1.919	1.595	0.103	0.226	0.214	0.023	0.033	0.034	0.964	1.114	1.085
11	0.240	0.206	0.212	1.540	2.891	2.624	0.085	0.177	0.165	0.024	0.034	0.034	0.760	0.843	0.827

^a^ Both = GFRP + CFRP.

**Table 11:** Stress–strain models of FRP-confined concrete.

Reference	Expression of stress–strain model	Model parameters
Lam and Teng [3]	$f_{c}=\{\begin{array}{c} E_{c}\varepsilon_{c}-\frac{{\left(E_{c}-E_{2}\right)}^{2}}{4f_{co}^{\prime }}\varepsilon_{c}^{2},\;0\leq \varepsilon_{c}\leq \varepsilon_{0}\\ f_{co}^{\prime }+E_{2}\varepsilon_{c},\;\varepsilon_{0}\leq \varepsilon_{c}\leq \varepsilon_{cu} \end{array}$	-
Harajli et al. [62]	$f_{c}=\{\begin{array}{c} f_{c1}\left[\frac{2\varepsilon_{c}}{\varepsilon_{c1}}-{\left(\frac{\varepsilon_{c}}{\varepsilon_{c1}}\right)}^{2}\right],0\leq \varepsilon_{c}\leq \varepsilon_{c1}\\ \sqrt{\left(k_{0}^{2}-k\right)}-k_{0},\varepsilon_{c1}\leq \varepsilon_{c}\leq \varepsilon_{cu} \end{array}$	$k_{0}=0.0031k_{1}E_{lf}-f_{co}$ $k_{1}=1.25{\left(\frac{f_{l}}{f_{co}}\right)}^{-0.5}$ $k={f_{co}}^{2}-0.0032k_{1}E_{lf}f_{co}\left(\frac{\varepsilon_{c}}{\varepsilon_{co}}+0.9\right)$
Youssef et al. [46]	$f_{c}=\{\begin{array}{c} E_{c}\varepsilon_{c}\left[1-\frac{1}{n}\left(1-\frac{E_{2}}{E_{c}}\right){\left(\frac{\varepsilon_{c}}{\varepsilon_{c1}}\right)}^{n-1}\right],0\leq \varepsilon_{c}\leq \varepsilon_{c1},E_{2}>0\\ E_{c}\varepsilon_{c}\left[1-\frac{1}{n}{\left(\frac{\varepsilon_{c}}{\varepsilon_{c1}}\right)}^{n-1}\right],\;0\leq \varepsilon_{c}\leq \varepsilon_{c1},E_{2}>0\\ f_{c1}+E_{2}\left(\varepsilon_{c}-\varepsilon_{c1}\right),\;\varepsilon_{c1}\leq \varepsilon_{c}\leq \varepsilon_{cu} \end{array}$	$n=\{\begin{array}{c} \frac{\left(E_{c}-E_{2}\right)\varepsilon_{c1}}{E_{c}\varepsilon_{c1}-f_{c1}},E_{2}>0\\ \frac{E_{c}\varepsilon_{c1}}{E_{c}\varepsilon_{c1}-f_{c1}},E_{2}<0 \end{array}$
Teng et al. [9]	$f_{c}=\{\begin{array}{c} E_{c}\varepsilon_{c}-\frac{{\left(E_{c}-E_{2}\right)}^{2}}{4f_{co}^{\prime }}\varepsilon_{c}^{2},\;0\leq \varepsilon_{c}\leq \varepsilon_{0}\\ f_{co}^{\prime }+E_{2}\varepsilon_{c},\;\varepsilon_{0}\leq \varepsilon_{c}\leq \varepsilon_{cu},if\;\rho_{k}\geq 0.01\\ f_{co}^{\prime }-\frac{f_{co}^{\prime }-f_{cu}}{\varepsilon_{cu}-\varepsilon_{co}}\left(\varepsilon_{c}-\varepsilon_{co}\right),\varepsilon_{0}\leq \varepsilon_{c}\leq \varepsilon_{cu},if\;\rho_{k}\leq 0.01 \end{array}$	
Wei and Wu [57]	$f_{c}=\{\begin{array}{c} E_{c}\varepsilon_{c}+\frac{f_{c1}-E_{c}\varepsilon_{c1}}{{\varepsilon_{c1}}^{2}}{\varepsilon_{c}}^{2},0\leq \varepsilon_{c}\leq \varepsilon_{c1}\\ f_{c1}+E_{2}\left(\varepsilon_{c}-\varepsilon_{c1}\right),\varepsilon_{c1}\leq \varepsilon_{c}\leq \varepsilon_{cu} \end{array}$	$E_{2}=\frac{f_{cu}-f_{c1}}{\varepsilon_{cu}-\varepsilon_{c1}}$
Samaan et al. [59]	$f_{c}=\frac{\left(E_{1}-E_{2}\right)\varepsilon_{c}}{{\left\{1+{\left[\frac{\left(E_{1}-E_{2}\right)\varepsilon_{c}}{f_{0}}\right]}^{n}\right\}}^{\frac{1}{n}}}+E_{2}\varepsilon_{c}$	$f_{0}=f_{cc}-E_{2}\varepsilon_{cu}$; $n=1+{\left(\frac{E_{1}}{E_{2}}-1\right)}^{-1}$
Zhou and Wu [61]	$\sigma =\left[\left(E_{1}\varepsilon_{n}-f_{0}\right)e^{-\frac{\varepsilon }{\varepsilon_{n}}}+f_{0}+E_{2}\varepsilon \right]\left(1-e^{-\frac{\varepsilon }{\varepsilon_{n}}}\right)$	$\varepsilon_{n}=f_{o}/E_{1}$; $E_{2}=\frac{f_{cc}-f_{o}}{\varepsilon_{cu}}$
Jiang and Teng [2]	$\frac{\sigma_{c}}{f_{cc}^{\prime *}}=\frac{\left(\varepsilon_{c}/\varepsilon_{cc}^{*}\right)r}{r-1+{\left(\varepsilon_{c}/\varepsilon_{cc}^{*}\right)}^{r}}$ $\begin{array}{c} \frac{\varepsilon_{c}}{\varepsilon_{co}}=0.85\left(1+8\frac{\sigma_{l}}{f_{co}^{\prime }}\right)\times {\{\left[1+0.75\left(\frac{-\varepsilon_{l}}{\varepsilon_{co}}\right)\right]}^{0.7}\\ -\exp\left[-7\left(\frac{-\varepsilon_{l}}{\varepsilon_{co}}\right)\right]\} \end{array}$	$r=\frac{E_{c}}{E_{c}-f_{cc}^{\prime *}/\varepsilon_{cc}^{*}}$; $\frac{f_{cc}^{\prime *}}{f_{co}^{\prime }}=1+3.5\frac{\sigma_{l}}{f_{co}^{\prime }}$ $\frac{\varepsilon_{cc}^{*}}{\varepsilon_{co}}=1+17.5{\left(\frac{\sigma_{l}}{f_{co}^{\prime }}\right)}^{1.2}$; $\sigma_{l}=-\frac{2E_{frp}t_{frp}\varepsilon_{l}}{D}$

## Notation

*E*_1_	initial elasticity modulus of FRP-confined concrete
*E*_2_	the slope of the asymptotical line of the hardening branch of the stress–strain curve
*E*_c_	initial elasticity modulus of concrete
*E_frp_*	elasticity modulus of FRP
*R*	the replacement ratio of RA
*D*	the diameter of the circular section
*f_frp_*	ultimate tensile strength of FRP
$f_{co}^{\prime }$	compressive strength of unconfined concrete
*f_cc_*	ultimate strength of CFRP-confined concrete
*k_ε_*	FRP strain efficiency factor
*f_o_*	stress value at the intersection of the back-extended asymptotical line of stress–strain curve
*n*	the shape parameter of the curve
*t_frp_*	thickness of FRP fiber
*ε_frp_*	ultimate strain of FRP
*ε_h,frp_*	average hoop rupture strain of CFRP
*ε_hm,frp_*	maximum hoop rupture strain of CFRP
*ε_o_*	axial peak strain
*ε_cu_*	ultimate strain of CFRP-confined concrete
